# Young-onset metastatic colorectal cancer: an opportunity and a vision for progress in cancer

**DOI:** 10.1007/s12032-025-02640-5

**Published:** 2025-03-08

**Authors:** Prasad D. Cooray, Nicole Jane Cooper

**Affiliations:** 1 Department of Surgery, University of Melbourne, Austin Health, Melbourne, VIC Australia; 2 Master of Business Administration, Melbourne, VIC Australia

**Keywords:** Young-onset colorectal cancer (yo-CRC), Metastatic colorectal cancer (mCRC), Personalised cancer treatment, Registry of Incidence, Intervention, and Outcomes (RIIO), Patient engagement, Adaptive longitudanal multidisciplinary care

## Abstract

Metastatic young-onset colorectal cancer (yo-CRC) is a distinct and aggressive disease subtype that is becoming increasingly prevalent worldwide with Australia leading the world in this trend. This article provides an evidence-based perspective, through the prism of authors’ personal experience, to craft an effective pathway not only to deliver improved outcomes for the patients but also to reduce disparities and foster collaboration amongst the cancer-treating community and indeed patients. It highlights an opportunity to re-define, re-design, and create a model that is rewarding to patients and cancer-treating community. Although our focus is on the high unmet needs group of yo-CRC, this model has the potential to expand to other cancer types and care models. We analyse the unique epidemiological trends, challenges, and burdens, emphasising the need for tailored treatment approaches for younger patients with colorectal cancer especially in the metastatic setting. We identify current gaps in clinical practice and research. To improve real-world outcomes, we propose a conceptual framework to enhance clinician–patient communication and treatment planning. Central to our approach is the integration of a Registry of Incidence, Intervention, and Outcomes (RIIO), which enables real-time data collection and analysis, improving treatment personalisation and efficacy. This registry could revolutionise patient care and drive research innovation through enhanced data sharing and collaboration. We advocate for a patient-centric integrated care model that utilises all available therapies to maximise survival and quality of life. Our perspective underscores the urgent need for a paradigm shift in how yo-CRC is viewed, researched and managed, proposing a pathway to significantly enhanced outcomes. Whilst it is feasible to expand the concepts discussed here for all colorectal cancer and indeed all cancer types, we believe this approach is most relevant and acutely needed in yo-CRC setting for reasons detailed in the manuscript.

“I was diagnosed with terminal bowel cancer when I was 32 years old. It came from nowhere and I was completely blindsided by cancer in my body. But what I found even more shocking than the cancer diagnosis itself, was the differing opinions on what my cancer diagnosis might mean, and what my prognosis might be … and I was shocked that all it took was another opinion, for someone to weigh in, not on the inevitability of my death, but on the potential and the opportunity for my life.”—Nicole Cooper, OAM (1984–2023).

Young-onset colorectal cancer (yo-CRC) refers to cases diagnosed in individuals under the age of 50. Globally, yo-CRC has been accelerating rapidly since mid-1990’s [1, 2]. Australia leads the world in this trend of yo-CRC incidence [3].

The causative factors specific to yo-CRC remain poorly defined. Whilst family history and inflammatory bowel disease are known risk factors, they account for only a small proportion of cases. The role of modifiable factors like obesity, red meat, and alcohol—established risks for late-onset CRC—is unclear in yo-CRC, likely due to shorter lifetime exposure [4–8].

Patients are more likely to be diagnosed at a later stage (metastatic yo-CRC) due to a variety of factors [9–13]. Emerging evidence also indicates distinct molecular profiles and more aggressive biology of yo-CRC compared to colorectal cancer (CRC) in older patients [14]. Metastatic cancer diagnosis in a young person carries with it an immense psycho-social and financial burden to the individual, their immediate family, and ultimately to the society at large [15].

Whilst early-stage CRC can be surgically cured with high survival rates, especially when combined with adjuvant and neoadjuvant therapies, the five-year overall survival rate for metastatic CRC (mCRC) stands at a dismal 13% [16–18]. Current survival data specific for metastatic yo-CRC are sparse. A population-based study in New South Wales (Australia) reported a five-year survival rate of 22.3% for the metastatic yo-CRC cohort, compared to 15.3% for older patients [19]. The most detailed analysis of survival outcomes is from the SEER database analysing mCRC patients from 2010 to 19 [20]. They found a statistically significant difference in median survival of 18 months for yo-CRC in comparison to 10 months for older patients. The authors postulated that this may be attributed to differences in treatment options, as young-onset cases often receive more radical intent treatments including surgery, radiotherapy, and chemotherapy and they supported the validity of such an approach in younger patients with colorectal cancer [20–22].

## A “survival gap” in metastatic colorectal cancer

In a seminal 2007 review, Goldberg et al., called for a shift in the treatment approach to mCRC, emphasising a move away from the traditional, sequential “lines” of therapy towards a more integrated, patient-centred continuum of care [23]. This entails a strategy where chemotherapy and biological treatments are adapted to the individual’s clinical situation, potentially including early switching of therapies, maintenance treatments, and surgical resection and local ablation of metastases when applicable. The underlying goal of this paradigm shift is to maximise patient exposure to all effective treatments, reduce unnecessary toxicity, and ultimately improve survival rates and quality of life for patients. This perspective is indeed more relevant today than ever, due to significant advances in systemic and biological therapies, innovations in targeted and immune therapies, surgical methods, and radiotherapy [24]. The broadened treatment options available necessitates ever more personalised treatment plans. Beyond any other group, this approach is particularly pertinent for metastatic yo-CRC patients to address the unique circumstances of this group.

The real-world application of this approach has been elegantly demonstrated in the recent Finnish RAXO studies [25, 26]. RAXO was a prospective, nationwide study of treatable mCRC patients of all age groups. The core principle was to follow individual patients’ disease trajectory in its entirety. Repeat and centralised assessments of resectability occurred during their systemic therapy. 37% of the patients had multiple metastasectomies for multisite or later developing metastasis. They demonstrated an impressive median overall survival of 80.4 months in the R0/R1 resected group, 39.1 months in the R2 resected or locally ablated group, and 20.8 months in the systemic therapy alone groups with 5-year overall survival rates of 66%, 40%, and 6%, respectively (R0/1/2 resections—see appendix).

The power of this study lies in its prospective nature, comprehensiveness of patient and data capture, and repeated intervention, demonstrating what is achievable in the real-world application of Goldberg et al.’s approach. Although there was no subgroup analysis of yo-CRC, by virtue of their better performance status and less comorbidities, one would expect that even better outcomes are feasible in this group, an observation also supported by the SEER study [20].

Therefore, what is clear is the existence of a “survival gap” in the domain of mCRC outcomes. This gap is the difference in what is achievable at best, with a coordinated and longitudinal continuum of care is applied versus the lowest common denominator possible with systemic therapy alone. It is important to understand the underlying factors contributing to this gap so that we can address it from a fresh perspective.

## The challenge of metastatic colorectal cancer

**Fig. 1:**
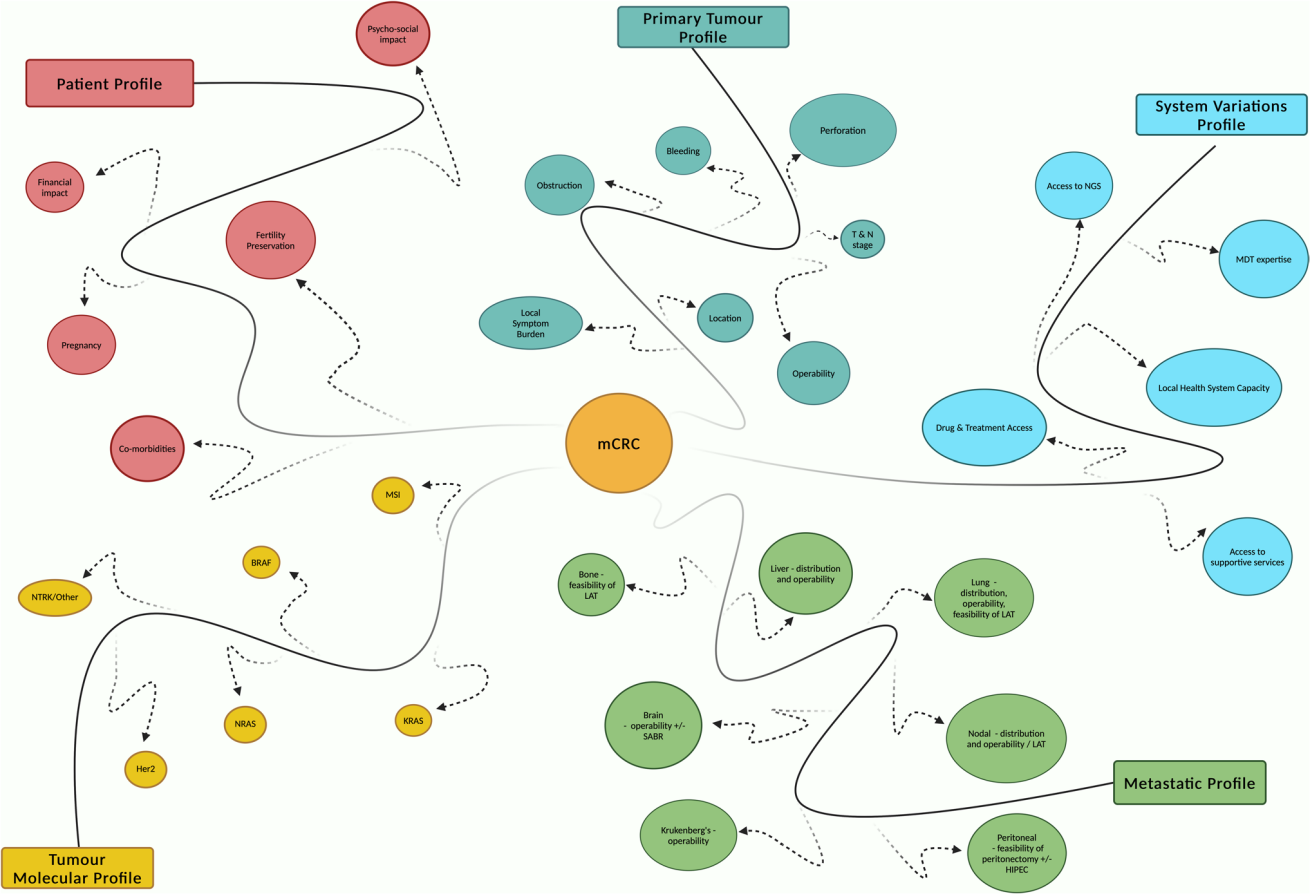
Complexities and Variables of Metastatic Colorectal Cancer. *HIPEC* Hyperthermic Intraperitoneal Chemotherapy, *LAT* Locally Ablative Therapies, *SABR* Stereotactic Ablative Radiation Therapy

Metastatic CRC is a complex and heterogeneous disease. Progress in CRC research and capabilities of therapeutic interventions underscores this complexity but nevertheless has created a multitude of interacting variables. There are variations in molecular profile, primary tumour profile, patient profile, and the metastatic profile unique to each patient as illustrated in Fig. 1. Based on these variables, the most suitable treatment is selected upfront, but each patient will have a unique and different signature disease trajectory during the course of their illness.

Whilst randomised controlled trials (RCTs) can address a single intervention or variable at a time, it is an insufficient tool to encompass and emulate the broad variations in multimodality treatment of mCRC in a continuum of care model during the entire disease trajectory. The reductionism view of RCTs, therefore, fails to support progress in the broader context of this now complex disease [27, 28].

Clinical practice guidelines are an endeavour to bridge this gap and aid decision-making in the clinic. By necessity, their reliance on RCTs, however, generates significant gaps in real-world applicability [29, 30]. The longitudinal view of variable disease trajectories is difficult to be protocolled for clinical application. This is relevant, as in the clinic one is navigating the entirety of a patient’s disease trajectory rather than simply deciding on the best first or second line therapy or best single intervention.

This difficulty of integrating longitudinal perspective into guidelines leads to limitations in clinical applications and hence variations and disparities in clinical outcomes. This limitation is indeed further complicated by the ongoing success in treatment advancements, which paradoxically introduces new challenges in therapeutic decision-making and practice implementation.

## A conceptual framework to progress patient care

Despite the challenges, metastatic yo-CRC presents significant opportunities for real-life application of the principles advocated by Goldberg et al. [23]. The majority of yo-CRC patients lack significant comorbidities and are motivated to achieve the best possible outcomes. Therefore this cohort avoids a number of variables that hinder optimal treatment in older patients.

In this context, an overarching objective of achieving substantial remission or cure for a large proportion of patients is not only appropriate but also feasible. This paradigm is underpinned by a “potential for life” philosophy. It necessitates an initial establishment of treatment intent and objectives in partnership with the patient. This critical step involves education and empowerment of patients, fostering a partnership that promotes self-advocacy and collaborative decision-making [31–33]. Such a foundation is indispensable to facilitate agility around treatment intent, objectives, and decision-making throughout the trajectory of the chronic illness [34, 35]. Indeed treatment intent requires the flexibility to be reviewed and reassessed at each step as patients navigate through the multimodality treatment landscape.

Rapid advancements in mCRC treatments over the past decade have significantly expanded the potential for cure for many patients. Whilst systemic therapies such as chemotherapy, biological agents, and immunotherapy have evolved, parallel progress in radiotherapy and surgical techniques has redefined what was once considered non-curable into potentially curable scenarios. However, outdated treatment intent definitions—“curative” and “palliative”—persist, limiting communication and clinical decision-making [36, 37]. These binary concepts, embedded in oncology practice and clinical trial design, fail to reflect the modern treatment spectrum. We propose a more flexible approach, categorising treatment intent as “radical” or “non-radical,” where radical intent encompasses multimodal therapies aimed at substantial remission or cure, whilst non-radical intent prioritises life extension with quality considerations. This approach allows for dynamic reassessment as patients respond to or progress after treatments, fostering open and adaptable conversations that align with evolving or diminishing therapeutic possibilities.

**Fig. 2:**
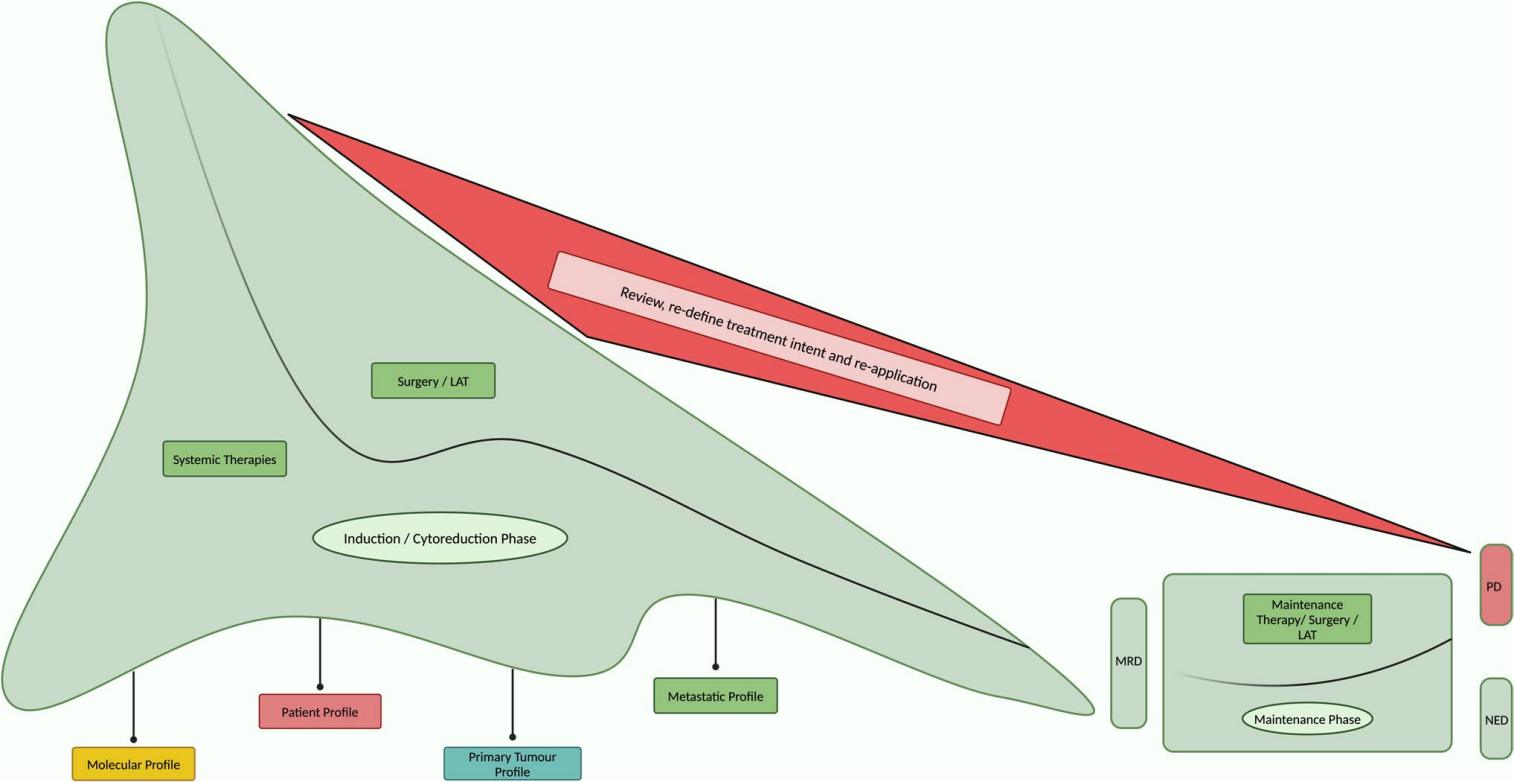
A Conceptual Framework to Optimise Outcomes for Metastatic Colorectal Cancer. *LAT* Locally Ablative Therapies, *MRD* Minimal Residual Disease, *NED* No Evidence of Disease, *PD* Progressive Disease

Our proposed framework for metastatic yo-CRC prioritises radical intent—aimed at achieving a substantial remission or indeed cure—as a primary approach for patients with metastatic yo-CRC (Fig. 2). Until recently the treatment paradigm for mCRC was dichotomised, at the time of diagnosis, to (a) resectable and (b) unresectable groups, with unresectable group designated for palliative treatment approaches. Clinical trials and therefore the evidence base is mostly reflective of this paradigm. Subsequently, more effective combinations of chemotherapy and biological therapies together with advances in surgical techniques evolved, leading to the emergence of a third category of (c) conversion therapy [38–42]. Conversion therapy has now been incorporated into ESMO Clinical Practice Guideline for mCRC treatment guidelines [29] in alignment with the continuum of care model.

The proposed framework entails

- An induction / cytoreduction phase utilising optimal systemic therapy to maximise depth of response (DoR) and/or conversion therapy to achieve resectability [43, 44].
- Intervention for the primary tumour and metastasectomies or locally ablative therapies (LAT) where appropriate, with a focus on attaining minimal residual disease (MRD).
- A maintenance phase, incorporating maintenance therapy, intervention for residual oligometastases, and enhanced monitoring using PET/CT/MRI imaging and potentially leveraging state-of-the-art technologies, such as circulating tumour DNA (ctDNA) analysis [45–50].
- A critical step of reviewing, re-defining treatment intent, and re-application phase at disease relapse or progression. As demonstrated in the RAXO study, many patients require multiple cycles of interventions to achieve the best outcome if not cure [25]. Therefore, close surveillance with re-defining treatment intent and re-application of the induction phase principles if appropriate at disease relapse or progression is an integral element of this paradigm.

We acknowledge that this framework may pose challenges depending on the expertise and resource availability of the healthcare system. Nevertheless a clearly defined framework supports adaptability, consistent and informed clinical decisions, communication and partnering with patients, and establishment of treatment intent and enhances the potential for achieving the best possible patient outcomes. It provides a visual representation of cancer treatment landscape which aids educating and empowering patients and demonstrates a focus on agility, regardless of the scopes and limitations of an individual healthcare system. Furthermore, it reflects the best practice standard of care in multi-disciplinary teams (MDTs) specialising in yo-CRC. Indeed it is a concept that has demonstrated efficacy in other cancer types where similar rapid advances have occurred, such as melanoma, breast, and lung cancer therapy.

## A pathway for research progress: national yo-CRC incidence, intervention, and outcomes registries

**Fig. 3:**
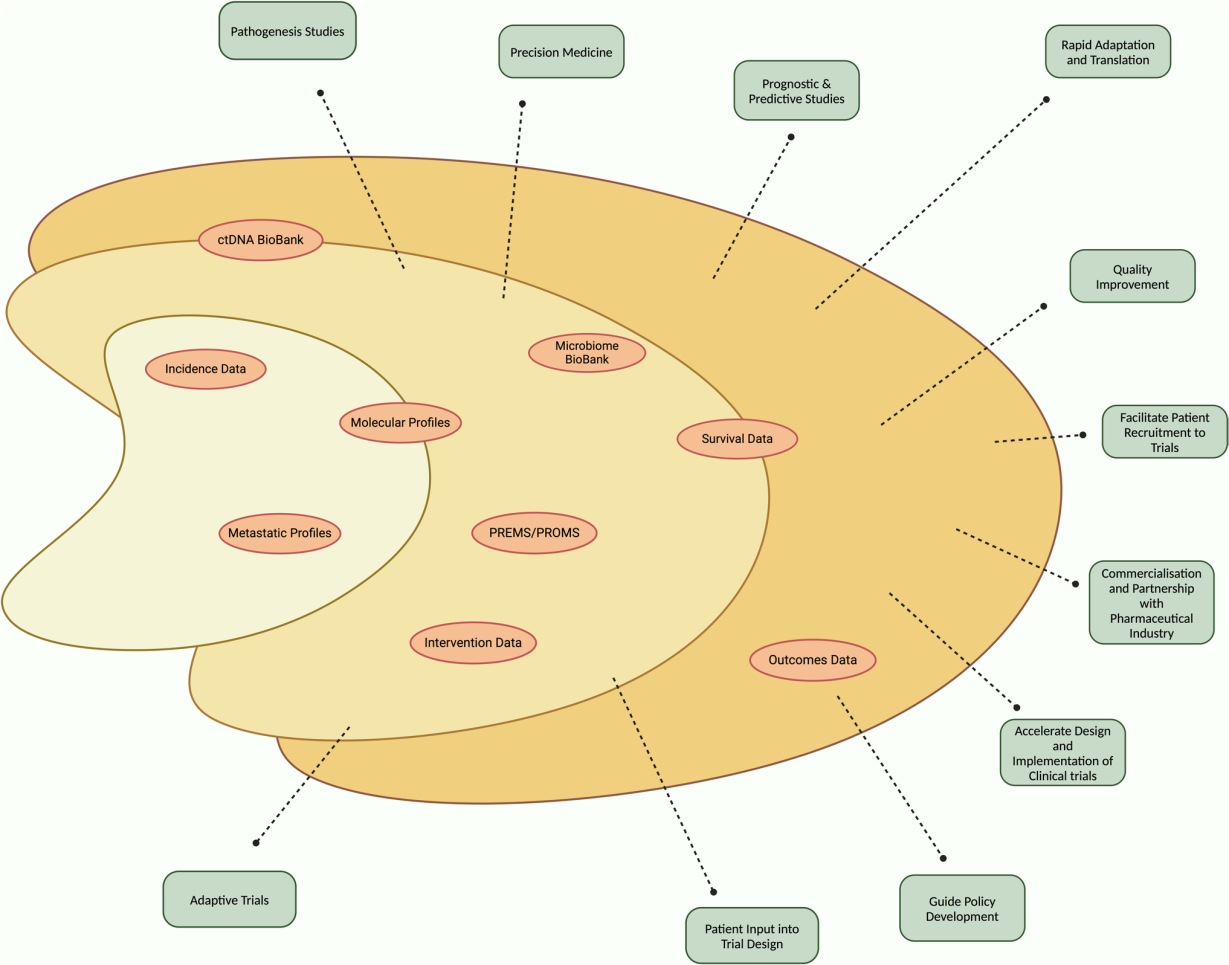
Components and Outputs of A Registry of Incidence Interventions and Outcomes (RIIO)

The centrepiece of this strategy is establishing a dedicated, real-time national registry—termed the Registry of Incidence, Intervention, and Outcomes (RIIO)—for metastatic yo-CRC patients. This registry will be instrumental in providing vital real-world outcomes data, enabling rapid adjustments to treatment protocols, and serving as a hub for comprehensive genomic data collection and bio-banking, including ctDNA and microbiome samples, thus facilitating current and future in-depth research into the disease’s biology (Fig. 3) [51, 52].

RIIO will also gather patient-reported outcome measures (PROMs) and patient-reported experience measures (PREMs), integrating patient perspectives into clinical trial design and ensuring timely access to trials [51, 53–56]. Additionally, the registry will include care navigators to address the complex challenges faced by yo-CRC patients in advanced stages of the disease [57, 58]. These navigators will play a crucial role, and it is essential that oncologists, with their deep expertise in CRC management, work closely and collaboratively with both the patient and the care navigator to optimise treatment outcomes.

Yuzhalin highlights a concerning trend of diminishing returns on significant investments in fundamental cancer research, exacerbated by a reductionist approach and the competitive nature of research funding, which often discourages the sharing of ideas and data [27]. To address these challenges, the establishment of a publicly funded, centralised, RIIO offers a strategic solution. This comprehensive RIIO could foster greater collaboration and data sharing, propelling us into the next generation of cancer research. Acting as a robust data reservoir, this registry would self-sustain research efforts. Focusing on a high-need area like metastatic yo-CRC, which has a relatively manageable cohort size—approximately 1,800 cases of all stages, annually in Australia—makes establishing such a registry feasible [59].

Existing nationwide cancer databases in Sweden, Japan, and Australia predominantly capture incidence and mortality data, which is a major drawback in their application for contemporary research needs. On the other hand the U.S. SEER database demonstrates a more detailed approach but lacks nationwide coverage. Australia’s smaller but more comprehensive databases and registries face challenges due to their limited scope, focus on specific aspects of the disease, and data ownership issues. Most registries do not have the capacity for real-time data capture thereby lacking the visibility needed to solve today’s problems—by being reliant on yesterday’s data.

Thus, a state-funded, comprehensive RIIO integrated with a biobank, targeting a small but underserved patient group, could significantly enhance and transform research in this field. This approach not only promises advancements in yo-CRC but also has the potential to broaden its applicability to other cancer types, substantially advancing the landscape of cancer research.

## A care model for metastatic yo-CRC

As demonstrated by the RAXO study, centralised surgical MDT assessments substantially assist the local MDTs [25]. This is eminently achievable via virtual MDT as demonstrated by the Finnish group and others [25, 59, 60]. Adopting the same technology used for virtual MDTs, it would be possible to allow the patients the option of attending their MDT’s thereby not only incorporating “patient voice” into the MDT but also enhance the transparency of the process where life-changing decisions are made for the patient [61, 62].

**Fig. 4:**
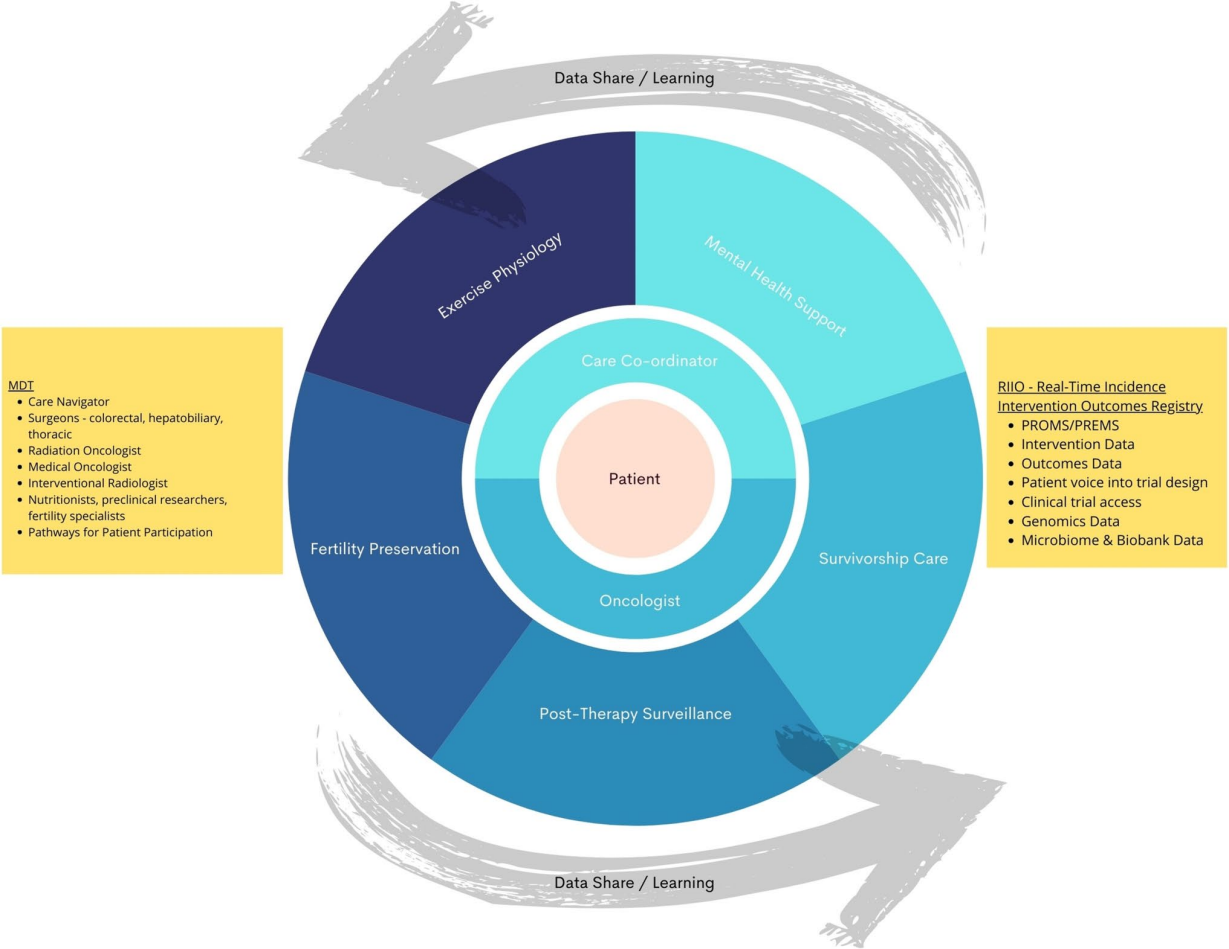
A Care Model for Young Onset Colorectal Cancer. *PREMS* Patient-Reported Experience Measures, *PROMS* Patient-Reported Outcome Measures

The local MDT designated for yo-CRC patients will comprise medical oncologists, radiation oncologists, surgeons, interventional radiologists, nutritionists, preclinical researchers, fertility specialists and care navigators. Care navigators could also bridge the potential communication gaps between patients, oncologists, and the RIIO. The collaborative efforts of oncologists and care navigators will extend to various facets of patient management, including fertility preservation, exercise physiology, mental health support, survivorship care, managing treatment-related complications, and diligent post-treatment surveillance (Fig. 4).

## Overcoming implementation challenges for a metastatic yo-CRC RIIO

Establishing the RIIO for metastatic yo-CRC presents significant challenges in funding, clinician engagement, stakeholder participation, and timely patient inclusion. We propose the following as a feasible strategy in the context of Australian healthcare system.

- Funding and Management: The RIIO would ideally be funded and managed under a large body, such as Medical Research Futures Fund (MRFF), which supports high-impact medical research and innovation [63, 64]. This aligns well with the MRFF’s objectives to enhance health outcomes by addressing national priorities. The registry will integrate a unique patient identifier (UPI) linked to each individual’s Medicare number at diagnosis, facilitating streamlined registration and accurate data capture through an online platform.
- Data Integrity and Compliance: Mandatory reporting to the RIIO at the time of histological confirmation, combined with a robust cross-referencing systems, will ensure the integrity and completeness of the registry. Linking the UPI with Medicare reimbursements could promote compliance, whilst integration with the Pharmaceutical Benefit Scheme will allow for precise treatment tracking.
- Educational and Incentive Programmes: Implementing RIIO necessitates a comprehensive educational campaign targeting clinicians, healthcare systems, and the public, complemented by a transparent patient consent process. Incentivising healthcare providers through linked Medicare reimbursements could further enhance participation and data quality.
- Addressing Challenges: The implementation strategy must consider potential system variations and is anticipated to be applicable across most developed healthcare systems. However, it requires meticulous planning to address data privacy and security, ensure stakeholder engagement, and manage financial and technological challenges. Ethical complexities, including informed consent, withdrawal of consent and incidental findings, must also be navigated carefully.

By addressing these challenges with anticipatory, flexible and thorough management strategies, the RIIO for yo-CRC can provide transformative insights and significantly advance patient and research outcomes.

## Conclusion

We hope to offer a perspective and a vision to managing metastatic yo-CRC, advocating for a shift to a dynamic, patient-centred continuum of care. This model emphasises early and when possible, radical intent multi-disciplinary interventions, integration of advanced therapeutic technologies, and ongoing patient engagement, specifically designed to meet the needs of yo-CRC patients. Despite potential challenges due to varying resources across different healthcare settings, the framework provides an adaptable roadmap for establishing clear treatment goals, enhancing patient-clinician partnerships, and enabling adaptive strategies to optimise outcomes. We seek a pathway through the establishment of comprehensive national incidence, intervention and outcomes registries together with care coordinators to address the disparities in metastatic yo-CRC, aiming for better outcomes and improved quality of life for younger patients affected by this condition. This approach has significant potential to bridge the knowledge gap by providing a holistic view and fostering collaboration to leap frog us to the next generation of cancer research.

## Data Availability

No datasets were generated or analysed during the current study.

## Appendix

R0 = surgical resection with microscopically negative margins

R1 = surgical resection with microscopically positive margins

R2 = incomplete surgical resection with macroscopically positive margins
